# The Turkish validation of the fullPIERS model to predict adverse maternal and fetal outcomes in preeclamptic pregnancies

**DOI:** 10.3389/fmed.2026.1847438

**Published:** 2026-06-29

**Authors:** Gokce Naz Kucukbas Ozonder, Arzu Yavuz, Emre Sertel, A. Seval Ozgu-Erdinc

**Affiliations:** 1 Perinatology Division, Obstetrics and Gynecology Department, Kocaeli City Hospital, Kocaeli, Türkiye; 2 Obstetrics and Gynecology Department, Kocaeli City Hospital, University of Health Sciences, Kocaeli, Türkiye; 3 Obstetrics and Gynecology Department, Derince Research and Training Hospital, University of Health Sciences, Kocaeli, Türkiye; 4 Perinatology Division, Obstetrics and Gynecology Department, Izmir City Hospital, University of Health Sciences, İzmir, Türkiye

**Keywords:** fullPIERS model, preeclampsia, pregnancy, Türkiye, validation

## Abstract

**Introduction:**

The fullPIERS *(The Preeclampsia Integrated Estimate of Risk)* model was developed and used in a prospective, multicenter study to identify women with preeclampsia at risk of developing complications within 7 days. This research aimed to elucidate the fullPIERS model and conduct its Turkish validation.

**Methods:**

A total of 214 women with singleton pregnancies complicated by preeclampsia at or before 40 weeks of gestation were retrospectively evaluated; 170 met the inclusion criteria and were included in the study. The diagnosis of preeclampsia was established in accordance with the diagnostic criteria outlined in the American College of Obstetricians and Gynecologists *(ACOG)* Practice Bulletin. FullPIERS scores were calculated using the online calculator available on the University of British Columbia Faculty of Medicine website.

**Results:**

There were statistically significant differences in gestational age at hospital admission and gestational age at delivery between patients who experienced maternal and/or fetal adverse events and those who did not (*p* < 0.05). There was also a significant difference in birth weight in combined maternal–fetal adverse event group and only maternal adverse event group (*p* = 0.001 and *p* < 0.05, respectively). There were differences in systolic blood pressure, creatinine, and AST values between those who experienced adverse events in mother and baby and those who did not *(p* < 0.001, *p* < 0.001, and *p* = 0.011, respectively). There were differences in systolic blood pressure, creatinine, AST, platelet count, proteinuria and oxygen saturation values between those who experienced only maternal adverse events and those who did not *(p* < 0.05, *p* < 0.001, *p* = 0.009, *p* < 0.05, *p* < 0.05 and *p* < 0.016 respectively). There was a significant difference in the fullPIERS score between those who experienced maternal, fetal, or combined maternal–fetal adverse events and those who did not (*p* < 0.001, *p* = 0.002 and *p* < 0.001, respectively).

**Discussion:**

The fullPIERS model captures key pathophysiological features of severe preeclampsia and demonstrates robust risk discrimination in a Turkish cohort in maternal and/or fetal adverse events, supporting its broader application in complication risk-oriented obstetric care.

## Introduction

Approximately 4.6% of all pregnancies are complicated by preeclampsia, a disorder characterized by new-onset hypertension and organ dysfunction occurring after the 20th week of gestation. Unfortunately, this proportion is expected to increase due to the rising maternal age and the increasing prevalence of conditions such as obesity, diabetes mellitus, and chronic hypertension. Preeclampsia is the second leading cause of maternal mortality worldwide and is among the five most common obstetric complications responsible for maternal deaths in Türkiye (1, 2).

Maternal complications of preeclampsia include cerebrovascular hemorrhage, pulmonary edema, acute renal failure, hepatic rupture, placental abruption, and eclampsia, all of which may result in maternal death. Fetal complications include intrauterine growth restriction, oligohydramnios, preterm delivery, and complications associated with prematurity (3). A predictive model capable of identifying which patients presenting with preeclampsia are at high risk for maternal-fetal complications would enable additional precautions in patient care and substantially reduce preeclampsia-related morbidity and mortality is still an unmet need. In other words, such a model could facilitate improved maternal and fetal outcomes through management strategies such as earlier timing of delivery and closer surveillance in preeclamptic pregnancies identified as high risk for adverse maternal-fetal outcomes.

The fullPIERS *(The Preeclampsia Integrated Estimate of Risk)* model, which has not previously been studied in Türkiye, was developed and used in a prospective, multicenter study to identify women with preeclampsia at risk of developing complications within 7 days; the study was published in The Lancet (4). The model incorporates gestational age, chest pain or dyspnea, oxygen saturation, platelet count, aspartate aminotransferase levels, and serum creatinine levels in its risk prediction algorithm. Although this internally validated model has not yet been externally validated at a tertiary care center in Türkiye, it represents a cost-effective tool for predicting adverse maternal-fetal outcomes associated with preeclampsia (5). Within the scope of this research, we aimed to elucidate the FullPIERS model and conduct its Turkish validation.

## Materials and methods

### Patients

A total of 214 women with singleton pregnancies complicated by preeclampsia at or before 40th gestational week were evaluated retrospectively. Multiple pregnancies involving twins or higher-order gestations; pregnancies that did not result in hospital delivery and therefore lacked adequate follow-up of maternal and fetal outcomes; pregnancies with uncertain gestational age due to inability to confirm dating; and pregnancies with missing data required for the model were excluded from the study. In addition, patients with chronic hypertension, diabetes mellitus, systemic lupus erythematosus, cardiovascular disease, superimposed preeclampsia, or sickle cell anemia, as well as pregnancies conceived via *in vitro* fertilization (IVF), were excluded. After this exclusion process, 170 women with singleton pregnancies complicated by preeclampsia were enrolled in this study.

### Ethical declaration

All procedures followed were in accordance with the ethical standards of the responsible committee on human experimentation *(institutional and national)* and with the Helsinki Declaration of 1975, as revised in 2008. Our institution has granted ethics committee approval under protocol number *2024–143*, and the local ethics committee has waived the requirement for informed consent due to the retrospective nature of the research.

### Diagnosis

The diagnosis of preeclampsia was established in accordance with the diagnostic criteria outlined in the American College of Obstetricians and Gynecologists *(ACOG)* Practice Bulletin. Accordingly, preeclampsia was defined as new-onset hypertension occurring after 20 weeks of gestation, characterized by a systolic blood pressure ≥140 mmHg and/or a diastolic blood pressure ≥90 mmHg on at least two occasions at least 4 h apart, in a previously normotensive woman, accompanied by proteinuria or, in the absence of proteinuria, evidence of end-organ dysfunction. Proteinuria was defined as ≥ 300 mg protein in a 24-h urine collection, a protein-to-creatinine ratio ≥ 0.3, or a urine dipstick reading of ≥ 1+. In the absence of proteinuria, preeclampsia was diagnosed based on the presence of one or more of the following features: thrombocytopenia *(platelet count < 100,000/μL)*, impaired liver function indicated by elevated liver transaminases to at least twice the upper limit of normal, new-onset renal insufficiency *(serum creatinine > 1.1 mg/dL or doubling of baseline creatinine)*, pulmonary edema, or new-onset cerebral or visual disturbances. Delivery was planned at 37 weeks of gestation or at the time of diagnosis after 37 weeks of gestation for women with preeclampsia without severe features, and at 34 weeks of gestation for those with severe features or at the time of diagnosis after 34 weeks of gestation who were eligible for expectant management. In women diagnosed with preeclampsia with severe features before 34 0/7 weeks of gestation, expectant management was pursued when maternal and fetal conditions remained stable, and continued until 34 weeks of gestation or until clinical deterioration occurred. These patients were closely monitored for maternal and fetal status, with serial laboratory evaluations including complete blood count with platelet count, liver function tests, and serum creatinine measurements.

The collected data included maternal age, gravidity, parity, number of living children, type of preeclampsia *(severe or non-severe)*, gestational age at hospitalization, the highest systolic and diastolic blood pressure values recorded on the first 2 days of admission, arterial oxygen saturation at presentation, presence of dyspnea or chest pain, highest serum aspartate aminotransferase *(AST)*, and serum creatinine levels and lowest platelet count on first 2 days, gestational age at delivery, given therapies such as magnesium, antenatal steroids, antihypertensive medication, maternal complications *(vaginal bleeding, headache, visual disturbances, cardiovascular, central nervous system, hematologic, hepatic, renal system, placental abruption)*, fetal and neonatal complications *(stillbirth, infant death)*, neonatal birth weight, Apgar scores, and duration of stay in the neonatal or adult intensive care unit. All variables included in the study were obtained as part of routine clinical care and were therefore available from existing medical records.

Adverse maternal outcomes were defined as the presence of one or more of the following conditions: severe central nervous system (CNS) involvement (Glasgow Coma Scale score < 13 or retinal detachment); cardiorespiratory complications (postoperative requirement for more than one antihypertensive agent, pulmonary edema, need for intubation, or oxygen saturation < 90%); hepatic involvement (liver enzyme levels elevated to more than twice the upper limit of normal); renal impairment (serum creatinine > 2.2 mg/dL); or hematological abnormalities (platelet count < 100,000/μL), as well as placental abruption, and hypocalcemia and rectus sheath hematoma.

Adverse fetal outcomes were defined as the occurrence of one or more of the following: intrauterine fetal demise during hospitalization, neonatal death, or admission to the neonatal intensive care unit.

FullPIERS scores were calculated using the online calculator available on the University of British Columbia Faculty of Medicine website.^1^

### Statistical analysis

Patient data collected within the scope of the study were analyzed using the IBM Statistical Package for the Social Sciences *(SPSS)* for Windows 30.0 *(IBM Corp., Armonk, NY).* To describe the variables of the study participants, frequency distribution tables were used for categorical variables; for continuous variables, means and standard deviations were reported for normally distributed data, and medians and interquartile ranges for non-normally distributed data. Continuous variables with a normal distribution were compared between pregnancies at high risk of complications according to the FullPIERS model, using Student’s *t*-test, whereas non-normally distributed variables were compared using the *Mann–Whitney U test.* Categorical variables were analyzed using the *chi-square test or Fisher’s exact* test, as appropriate. The results were considered statistically significant when the *p*-value was less than 0.05.

The predictive performance of the fullPIERS model for complication risk was evaluated using receiver operating characteristic *(ROC)* curve analysis with a 95% confidence interval. Sensitivity, specificity, positive and negative predictive values, and positive and negative likelihood ratios were calculated using the determined cutoff value.

Calibration of the model for maternal, fetal, and combined maternal–fetal adverse events at 48 h and 7 days was evaluated using the Hosmer–Lemeshow goodness-of-fit test, demonstrating acceptable model fit for all outcomes.

Decision curve analysis (DCA) was performed to evaluate the clinical utility of the fullPIERS score for predicting maternal, fetal and combined adverse events within 48 h and 7 days. The decision curve analysis performed by using the “dcurves” package in R.

## Results

**FIGURE 1:**
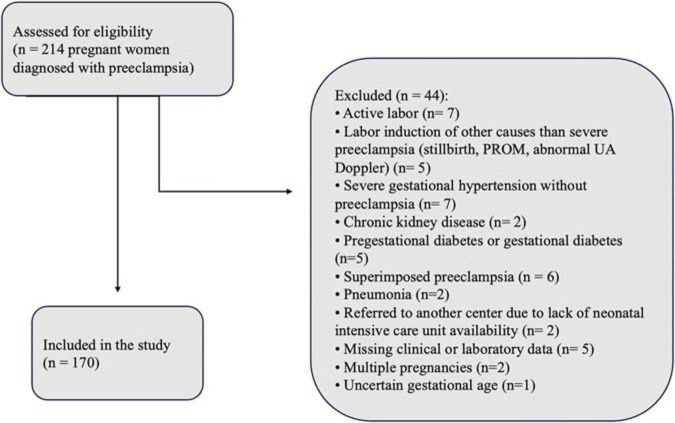
Study flowchart illustrating patient screening, eligibility assessment, exclusion criteria, and final cohort inclusion.

A total of 170 pregnancies complicated with preeclampsia were included in this study. The patient selection process is shown in Figure 1. The main characteristics of the participants are given in Table 1. More than three quarters of patients had preeclampsia with severe features. The average gestational age at admission was 34 weeks, and at delivery was 35 weeks. Half of the pregnancies were delivered within 2 days, and 80% within a week. Maternal and neonatal outcomes, including the highest maternal blood pressure values during the first 2 days of admission, proteinuria, systemic involvement, adverse events, birth weight, Apgar scores, and neonatal intensive care unit admission rates, are presented in Table 1.

**TABLE 1:** Main characteristics of the preeclamptic pregnancies participated in this study.

Parameters	Mean ± SD median (min-max)
Maternal age (year)	31.3 ± 6.4
Gravida (n)	2 (1–6)
Parity (n)	0 (0–5)
Gestational age at hospital admission (days)	241.5 (177–282)
Gestational age at time of delivery (days)	246 (177–282)
Duration from admission to delivery (days)	2 (0–67)
Maternal highest systolic BP (mmHg)	158.8 ± 13.47
Maternal highest diastolic BP (mmHg)	92.2 ± 10.81
Urine protein/creatinine ratio (mg/kg creatinine)	707 (52–30,372)
24-h urinary protein (mg/day)	707 (60–23,421)
Birth weight (gr)	2207.7 ± 856.6
1 min Apgar score	8 (0–9)
5 min Apgar score	9 (0–10)
	**n (%)**
Severe preeclampsia	150 (88.2%)
NICU admission	83 (48.8%)
Fetal/neonatal demise	7 (4.1%)
Maternal CNS involvement	3 (1.8%)
Maternal CVS involvement	49 (28.8%)
Maternal hematologic involvement	16 (9.4%)
Maternal hepatic involvement	12 (7.1%)
Maternal renal involvement	3 (1.8%)
Placental abruption	8 (4.7%)
Maternal ICU admission	9 (5.3%)
Combined adverse event	99 (58.2%)
Maternal adverse event	73 (43.2%)
Fetal/neonatal adverse event	84 (49.4%)
Delivery in 48 h	93 (54.7%)
Delivery in 7 days	137 (80.6%)
Delivery after 7 days	33 (19.4%)

BP, blood pressure; min, minute; NICU, neonatal intensive care unit; CNS, central nervous system; CVS, cardiovascular system; ICU, intensive care unit; Combined adverse event, maternal and fetal adverse events occurring together.

### Analysis of fullPIERS scores

The mean and median values of all parameters contributing to the fullPIERS score in the study population are presented in Table 2. Median fullPIERS scores according to delivery timing are also provided at the end of Table 2 for deliveries within 48 h, within 7 days, and after 7 days.

**TABLE 2:** Parameters of the fullPIERS score, overall scores in preeclamptic pregnancies, and scores stratified by delivery timing.

Parameters	n (%)
Chest pain/dyspnea	7 (4.1%)
	**Mean ± SD** **Median (min-max)**
O_2_ saturation (%)	96.8 ± 1.41
Platelet count (10^3^/ μL)	233 (77–449)
Creatinine (mg/dL)	0.57 ± 0.13
AST (U/L)	21 (9–464)
FullPIERS score (n)	0.7 (0–89.5)
FullPIERS score of pregnancies delivered in 2 days (n)	0.7 (0–89.5)
FullPIERS score of pregnancies delivered in 7 days (n)	0.6 (0–89.5)
FullPIERS score of pregnancies delivered after 7 days (n)	0.7 (0.1–4.9)

O_2_, oxygen; AST, aspartate aminotransferase.

FullPIERS scores were further analyzed according to the presence of adverse events. The scores for pregnancies complicated by combined maternal–fetal adverse events, fetal adverse events only, and maternal adverse events only are presented in Table 3. In the same table, fullPIERS scores were also compared between pregnancies with and without adverse events. Pregnancies complicated by combined maternal–fetal, fetal-only, and maternal-only adverse events had significantly higher fullPIERS scores compared with pregnancies without adverse events (*p* < 0.001, *p* = 0.002, and *p* < 0.001, respectively).

**TABLE 3:** Comparison of FullPIERS scores between patients with and without maternal, fetal, or combined adverse events.

Adverse event	No	Yes	*p*
Maternal and fetal	0.45 (0.10–2.90)	0.90 (0–89.50)	<0.001
Fetal	0.50 (0.10–89.50)	0.85 (0–87.80)	0.002
Maternal	0.50 (0–4.30)	1.00 (0.10–89.50)	<0.001

FullPIERS scores of pregnancies delivered within 48 hours and within 7 days are presented in Table 4. A subgroup analysis including pregnancies below 37 weeks of gestation was also performed according to delivery timing. No significant differences were observed in fullPIERS scores according to delivery timing either in the overall study population or among pregnancies below 37 weeks of gestation (Table 5).

**TABLE 4:** Comparison of fullPIERS scores according to delivery timing.

Delivery	Yes	No	*p*
FullPIERS score 48 h	0.7 (0–89.5)	0.7 (0.10–39.50)	0.668
FullPIERS score 7d	0.6 (0–89.5)	0.7 (0.10–4.9)	0.627

FullPIERS score 48 h: fullPIERS score of pregnancies delivered in 48 h, fullPIERS score 7d: fullPIERS score of pregnancies delivered in 7 days.

**TABLE 5:** Comparison of fullPIERS scores according to delivery timing in preeclamptic pregnancies before 37 weeks of gestation (subgroup analysis).

Delivery	Yes	No	*p*
fullPIERS score 48 h	0.80 (0–89.5)	0.70 (0.1–39.5)	0.979
fullPIERS score 7 d	0.70 (0–89.50)	0.70 (0.10–4.90)	0.912

### Combined maternal and fetal adverse outcomes

There were statistically significant differences between those who experienced adverse events in mother and baby and those who did not in terms of *“gestational age at hospital admission”* and *“gestational age at birth” (p < 0.001)*. Both durations were shorter in those who experienced adverse events in the mother and baby. There was also a significant difference in birth weight (*p* = 0.001). Birth weight was significantly lower in those who experienced adverse events. There were differences in systolic blood pressure, creatinine, and AST values between those who experienced adverse events in mother and baby and those who did not *(p* < 0.001, *p* < 0.001, *and p* = 0.011, respectively). These values were significantly higher in those who experienced adverse events. There was a significant difference in the fullPIERS score between those who experienced adverse events in the mother and baby and those who did not (*p* < 0.001). This score was higher in those who experienced adverse events. There were no significant differences in other variables between those who experienced adverse events in the mother and baby and those who did not (Table 6.1).

**TABLE 6.1:** Comparison between characteristics of pregnancies with and without combined adverse events.

Parameters	Presence of combined adverse event		*p*
	No	Yes	
Maternal age (years)	30.7 ± 6.8 31 (19–43)	31.7 ± 6.1 32 (16–44)	0.276^a^
Gestational age at hospital admission (days)	245.6 ± 21.1 253 (180–282)	227.7 ± 23.0 227 (177–277)	**<0.001^a^**
Gestational age at delivery (days)	251.2 ± 17.1 257 (181–282)	233.1 ± 21.6 236 (177–277)	**<0.001^a^**
Duration from admission to delivery (days)	5.6 ± 10.6 1 (0–67)	5.4 ± 9.2 2 (0–42)	0.466^a^
Maternal highest systolic BP (mmHg)	154.7 ± 11.2 151 (140–181)	161.7 ± 14.2 160 (138–201)	**0.001^a^**
Maternal highest diastolic BP (mmHg)	91.1 ± 10.8 90 (60–116)	92.9 ± 10.8 94 (57–120)	0.296^a^
Urine protein/creatinine ratio (mg/kg creatinine)	2008.7 ± 3641.3 548 (88–20,375)	3025.5 ± 4905.9 815.5 (52–30,372)	0.147^a^
24-h urinary protein (mg/day)	1870.9 ± 3613.0 507 (72–23,421)	2525.0 ± 3337.9 867 (60–16,587)	0.049^a^
Birth weight (gr)	2422.4 ± 764.4 2,550 (710–3,830)	2053.6 ± 889.2 1,886 (480–5,670)	**0.001^a^**
1 min Apgar score	7.2 ± 1.5 8 (0–9)	7.1 ± 1.4 8 (0–8)	0.275^a^
5 min Apgar score	8.4 ± 1.5 9 (0–10)	8.3 ± 1.5 9 (0–10)	0.344^a^
Gravida (n)	1 (1–6)	2 (1–6)	0.191^a^
Parity (n)	0 (0–5)	1 (0–5)	0.213^a^
Platelet count (10^3^/ μL)	246.9 ± 66.5 233 (125–449)	229.3 ± 81.4 220 (77–428)	0.119^a^
Creatinine (mg/dL)	0.52 ± 0.11 0.50 (0.30–0.83)	0.61 ± 0.14 0.61 (0.3–1.0)	**< 0.001^b^**
AST (U/L)	22.2 ± 13.4 19 (10–100)	32.2 ± 49.4 22 (9–464)	**0.011^a^**
O_2_ saturation (%)	97.0 ± 0.16 97 (96–98)	96.6 ± 1.82 97 (85–99)	0.418^a^
fullPIERS score	0.62 ± 0.55 0.45 (0.10–2.90)	4.5 ± 13.8 0.90 (0–89.5)	**< 0.001^a^**

^a^Mann Whitney U test;

^b^Independent samples *t*-test. BP, blood pressure; min, minute; AST, aspartate aminotransferase; O_2_, oxygen. Bold values indicate statistical significance.

There were statistically significant differences (*p* < 0.05) between those who experienced adverse events in the mother and baby and those who did not, in terms of preeclampsia type, antenatal steroids, antihypertensive, maternal complications, cardiovascular events, hematological events, hepatic events, placental events, and other adverse events (hypocalcemia and rectus sheath hematoma). The rate of adverse events in mother and baby was significantly higher in those with severe preeclampsia and those administered antenatal steroids and using antihypertensive drugs. Adverse events in mothers and babies were significantly lower among those with placental events. There were no significant differences between those who experienced adverse events in the mother and baby and those who did not, with respect to other variables (Table 6.2).

**TABLE 6.2:** Comparison between characteristics of pregnancies with and without combined adverse events (continued).

Parameters		Presence of combined adverse event		*p*
		No	Yes	
Severe preeclampsia	Yes	57 (38.3%)	92 (61.7%)	**0.042^a^**
	No	13 (65.0%)	7 (35.0%)	
Magnesium prophylaxis	No	16 (53.3%)	14 (46.7%)	0.226^a^
	Yes	55 (39.3%)	85 (60.7%)	
Antenatal steroid prophylaxis	No	51 (54.3%)	43 (45.7%)	**<0.001^b^**
	Yes	20 (26.3%)	56 (73.7%)	
Antihypertensive use	No	49 (52.1%)	45 (47.9%)	**0.002^b^**
	Yes	22 (28.9%)	54 (71.1%)	
Vaginal bleeding	No	69 (42.3%)	94 (57.7%)	0.701^c^
	Yes	2 (28.6%)	5 (71.4%)	
Abdominal pain	No	67 (44.1%)	85 (55.9%)	0.127^a^
	Yes	4 (22.2%)	14 (77.8%)	
Headache/visual disturbance	No	33 (43.4%)	43 (56.6%)	0.694^b^
	Yes	38 (40.4%)	56 (59.6%)	
Proteinuria	No	15 (42.9%)	20 (57.1%)	1.000^b^
	Yes	56 (41.5%)	79 (58.5%)	
Dipstick proteinuria	No	14 (42.4%)	19 (57.6%)	1.000^a^
	Yes	57 (41.6%)	80 (58.4%)	
Maternal complication	No	70 (46.4%)	81 (53.6%)	**0.003^a^**
	Yes	1 (5.9%)	16 (94.1%)	
Fetal complication	No	70 (44.0%)	89 (56.0%)	0.080^c^
	Yes	2 (11.1%)	8 (88.9%)	
Maternal CNS involvement	No	71 (42.5%)	96 (57.5%)	0.266^c^
	Yes	0 (0%)	3 (100.0%)	
Maternal CVS involvement	No	71 (58.7%)	50 (41.3%)	**< 0.001^a^**
	Yes	0 (0%)	49 (100.0%)	
Maternal hematologic involvement	No	71 (46.1%)	83 (53.9%)	**0.001^a^**
	Yes	0 (0%)	16 (100%)	
Maternal hepatic involvement	No	71 (44.9%)	87 (55.1%)	**0.006^a^**
	Yes	0 (0%)	12 (100%)	
Maternal renal involvement	No	71 (42.5%)	96 (57.5%)	0.266^a^
	Yes	0 (0%)	3 (100%)	
Placental abruption	No	64 (39.5%)	98 (60.5%)	**0.010^c^**
	Yes	7 (87.5%)	1 (12.5%)	
Other involvement	No	71 (44.9%)	87 (55.1%)	**0.006^a^**
	Yes	0 (0%)	12 (100%)	

^a^Yates chi-square test;

^b^Pearson chi-square test;

^c^Fisher chi-square test. CNS, central nervous system; CVS, cardiovascular system. Bold values indicate statistical significance.

There was a significant difference between those who experienced adverse events in both mother and baby and those who did not in terms of neonatal ICU admission and the presence of chest pain (*p* < 0.001 and *p* = 0.042). Similarly, the rate of adverse events in both mother and baby was significantly higher in those admitted to the neonatal ICU. Likewise, the rate of adverse events in both mother and baby was significantly higher in those experiencing chest pain. There were no significant differences in other variables between those who experienced adverse events in the mother and baby and those who did not (Table 6.3).

**TABLE 6.3:** Comparison between characteristics of pregnancies with and without combined adverse events (continued).

Parameters		Presence of combined adverse event		*p*
		No	Yes	
Maternal ICU admission	No	70 (43.5%)	91 (56.5%)	0.081^a^
	Yes	1 (11.1%)	8 (88.9%)	
NICU admission	No	59 (67.8%)	28 (32.2%)	**< 0.001^b^**
	Yes	12 (14.5%)	71 (85.5%)	
Fetal death	No	71 (42.3%)	97 (57.7%)	0.511^a^
	Yes	0 (0%)	2 (100%)	
Infant death	No	71 (43.0%)	94 (57.0%)	0.076^a^
	Yes	0 (0%)	5 (100%)	
Delivery in 48 h	No	31 (40.3%)	46 (59.7%)	0.717^c^
	Yes	40 (43.0%)	53 (57.0%)	
Delivery in 7 days	No	15 (45.5%)	18 (54.5%)	0.778^b^
	Yes	56 (40.9%)	81 (59.1%)	
Delivery after 7 days	No	56 (40.9%)	81 (59.1%)	0.778^b^
	Yes	15 (45.5%)	18 (54.5%)	
Chest pain	No	71 (43.6%)	92 (56.4%)	**0.042^a^**
	Yes	0 (0%)	7 (100%)	

^a^Fisher chi-square test;

^b^Yates chi-square test;

^c^Pearson chi-square test. ICU, intensive care unit; NICU, neonatal intensive care unit. Bold values indicate statistical significance.

### Fetal adverse outcomes

There were statistically significant differences between infants who experienced adverse events and those who did not in terms of *“gestational age at hospital admission,” “gestational age at delivery,” and “time to delivery” (p* < 0.001; *p* < 0.001, and *p* = 0.027, respectively). Gestational age at hospital admission and gestational age at delivery were significantly lower in infants who experienced adverse events. The time to delivery was significantly higher in infants who experienced adverse events.

There were differences between infants who experienced adverse events and those who did not in terms of maternal systolic blood pressure, 24-h proteinuria, creatinine, and aspartate transaminase values (*p* = 0.004; *p* = 0.035; *p* = 0.022; and *p* = 0.010). These values were significantly higher in mothers of infants who experienced adverse events.

There is a significant difference between infants who experienced adverse events and those who did not in terms of the fullPIERS score (*p* = 0.002). This score was higher in infants who experienced adverse events. There were no significant differences between infants who experienced adverse events and those who did not with respect to other variables (Table 7.1).

**TABLE 7.1:** Comparison between characteristics of pregnancies with and without fetal adverse events.

Parameters	Presence of fetal adverse event		*p*
	No	Yes	
Maternal age (years)	30.8 ± 6.4 31 (19–43)	31.8 ± 6.4 32 (16–44)	0.296^a^
Gestational age at hospital admission (days)	251.0 ± 14.59 253 (192–282)	219.0 ± 20.4 218.5 (177–269)	**< 0.001^a^**
Gestational age at delivery (days)	255.2 ± 10.34 257.5 (229–1‘282)	225.8 ± 20.2 228 (177–269)	**<0.001^a^**
Duration from admission to delivery (days)	4.2 ± 9.2 1 (0–67)	6.8 ± 10.3 2.5 (0–42)	**0.027^a^**
Maternal highest systolic BP (mmHg)	155.7 ± 11.9 153 (140–201)	161.9 ± 14.3 160 (138–200)	**0.004^a^**
Maternal highest diastolic BP (mmHg)	91.3 ± 10.9 90 (60–116)	93.1 ± 10.7 92.5 (57–120)	0.382^a^
Urine protein/creatinine ratio (mg/kg creatinine)	1665.5 ± 2688.1 610.5 (88–14455)	3556.6 ± 5553.6 901 (52–30372)	0.068^a^
24-h urinary protein (mg/day)	1581.2 ± 2514.7 556 (72–13726)	2924.9 ± 4103.9 1003 (60–23421)	**0.035^a^**
Birth weight (gr)	2243.4 ± 845.4 2315 (480–4175)	2171.1 ± 871.4 2165 (695–5670)	0.382^a^
1 min Apgar score	7.1 ± 1.5 8 (0–9)	7.2 ± 1.4 8 (0–8)	0.368^a^
5 min Apgar score	8.3 ± 1.5 9 (0–10)	8.4 ± 1.5 9 (0–10)	0.570^a^
Gravida (n)	2 (1–6)	2 (1–6)	0.330^a^
Parity (n)	0 (0–5)	0.5 (0–5)	0.535^a^
Platelet count (10^3^/ μL)	243.4 ± 72.5 242 (77–449)	229.8 ± 78.9 219 (82–428)	0.159^a^
Creatinine (mg/dL)	0.55 ± 0.12 0.54 (0.3–0.9)	0.60 ± 0.14 0.6 (0.3–1.0)	**0.022^b^**
AST (U/L)	21.8 ± 11.9 20 (10–100)	34.3 ± 53.4 22.5 (9–464)	**0.010^a^**
O_2_ saturation (%)	96.7 ± 1.24 97 (88–98)	96.8 ± 1.57 97 (85–99)	0.185^a^
fullPIERS score	2.56 ± 10.7 0.50 (0.1–89.5)	3.28 ± 10.91 0.85 (0–87.8)	**0.002^a^**

^a^Mann Whitney U test;

^b^Independent samples *t*-test. BP, blood pressure; min, minute; AST, aspartate aminotransferase,;O_2_, oxygen. Bold values indicate statistical significance.

There were statistically significant differences between those who experienced adverse infant events and those who did not in terms of preeclampsia type, antenatal steroids, antihypertensive, fetal complications, and cardiovascular events (*p* < 0.05). The rate of adverse infant events was significantly higher in those with severe preeclampsia. The rate of adverse infant outcomes was significantly higher in patients who received antenatal corticosteroids and antihypertensive therapy. The rate of adverse infant events was significantly higher in the group with fetal complications. The rate of adverse infant events was significantly higher in those with maternal cardiovascular events. There were no significant differences between those who experienced adverse infant events and those who did not across other variables (Table 7.2).

**TABLE 7.2:** Comparison between characteristics of pregnancies with and without fetal adverse events (continued).

Parameters		Presence of fetal adverse event		*P*
		No	Yes	
Severe preeclampsia	Yes	70 (47.0%)	79 (53.0%)	**0.034^a^**
	No	15 (75.0%)	5 (25.0%)	
Magnesium prophylaxis	No	16 (53.3%)	14 (46.7%)	0.896^a^
	Yes	70 (50.0%)	70 (50.0%)	
Antenatal steroid prophylaxis	No	68 (72.3%)	26 (27.7%)	**<0.001^a^**
	Yes	18 (23.7%)	58 (76.3%)	
Antihypertensive use	No	63 (67.0%)	31 (33.0%)	**<0.001^b^**
	Yes	23 (30.3%)	53 (69.7%)	
Vaginal bleeding	No	85 (52.1%)	78 (47.9%)	0.062^c^
	Yes	1 (14.3%)	6 (85.7%)	
Abdominal pain	No	81 (53.3%)	71 (46.7%)	0.072^a^
	Yes	5 (27.8%)	13 (72.2%)	
Headache/visual disturbance	No	38 (50.0%)	38 (50.0%)	0.890^b^
	Yes	48 (51.1%)	46 (48.9%)	
Proteinuria	No	19 (54.3%)	16 (45.7%)	0.763^a^
	Yes	67 (49.6%)	68 (50.4%)	
Dipstick proteinuria	No	16 (48.5%)	17 (51.5%)	0.940^a^
	Yes	70 (51.1%)	67 (48.9%)	
Maternal complication	No	80 (53.0%)	71 (47.0%)	0.113^a^
	Yes	5 (29.4%)	12 (70.6%)	
Fetal complication	No	85 (53.5%)	74 (46.5%)	**0.001^c^**
	Yes	0 (0%)	9 (100%)	
Maternal CNS involvement	No	86 (51.5%)	81 (48.5%)	0.118^c^
	Yes	0 (0%)	3 (100%)	
Maternal CVS involvement	No	68 (56.2%)	53 (43.8%)	**0.033^a^**
	Yes	18 (36.7%)	31 (63.3%)	
Maternal hematologic involvement	No	80 (51.9%)	74 (48.1%)	0.402^a^
	Yes	6 (37.5%)	10 (62.5%)	
Maternal hepatic involvement	No	81 (51.3%)	77 (48.7%)	0.733^a^
	Yes	5 (41.7%)	7 (58.3%)	
Maternal renal involvement	No	85 (50.9%)	82 (49.1%)	0.618^c^
	Yes	1 (33.3%)	2 (66.7%)	
Placental abruption	No	80 (49.4%)	82 (50.6%)	0.278^c^
	Yes	6 (75.0%)	2 (25.0%)	
Other involvement	No	82 (51.9%)	76 (48.1%)	0.347^a^
	Yes	4 (33.3%)	8 (66.7%)	

^a^Yates chi-square test;

^b^Pearson chi-square test;

^c^Fisher chi-square test. CNS, central nervous system; CVS, cardiovascular system. Bold values indicate statistical significance.

There were statistically significant differences between those who experienced adverse events in their infants and those who did not in terms of maternal ICU admission, neonatal ICU admission, and infant mortality (*p* = 0.017, *p* < 0.001, and *p* = 0.028). The rate of adverse events in infants was significantly higher in those who received maternal ICU and neonatal ICU admission. Similarly, the rate of adverse events in infants was significantly higher in those who died from infant mortality. There were no significant differences between those who experienced adverse events in infants and those who did not, with respect to other variables (Table 7.3).

**TABLE 7.3:** Comparison between characteristics of pregnancies with and without fetal adverse events (continued).

Parameters		Presence of fetal adverse event		
		No	Yes	*p*
Maternal ICU admission	No	85 (52.8%)	76 (47.2%)	**0.017^a^**
	Yes	1 (11.1%)	8 (88.9%)	
NICU admission	No	86 (98.9%)	1 (1.1%)	**<0.001^b^**
	Yes	0 (0%)	83 (100%)	
Fetal death	No	86 (51.2%)	82 (48.8%)	0.243^a^
	Yes	0 (0%)	2 (100%)	
Infant death	No	86 (52.1%)	79 (47.9%)	**0.028^a^**
	Yes	0 (0%)	5 (100%)	
Delivery in 48 h	No	35 (45.5%)	42 (54.5%)	0.223^c^
	Yes	51 (54.8%)	42 (45.2%)	
Delivery in 7 days	No	12 (36.4%)	21 (63.6%)	0.104^b^
	Yes	74 (54.0%)	63 (46.0%)	
Delivery after 7 days	No	74 (54.0%)	63 (46.0%)	0.104^b^
	Yes	12 (36.4%)	21 (63.6%)	
Chest pain	No	83 (50.9%)	80 (49.1%)	0.718^a^
	Yes	3 (42.9%)	4 (57.1%)	

^a^Fisher chi-square test;

^b^Yates chi-square test;

^c^Pearson chi-square test. ICU, intensive care unit; NICU, neonatal intensive care unit. Bold values indicate statistical significance.

### Maternal adverse outcomes

There were statistically significant differences between those who experienced adverse events in their mothers and those who did not in terms o*f “gestational age at hospital admission” and “gestational age at birth” (p* = 0.022; and *p* = 0.002). Both durations were shorter in those who experienced adverse events in their mothers. There was also a significant difference in birth weight (*p* = 0.028). Birth weight was significantly lower in those who experienced adverse events.

There were significant differences in systolic blood pressure, spot, and 24-h proteinuria, PLT, creatinine, AST, and O_2_ saturation between those who experienced maternal adverse events and those who did not (*p* < 0.05). In those who experienced adverse events, systolic blood pressure, spot, and 24-h proteinuria, creatinine, and AST values were significantly higher, platelet and oxygen saturation were significantly lower. There was a significant difference in the fullPIERS score between those who experienced maternal adverse events and those who did not (*p* < 0.001). The score was higher in those who experienced adverse events. There were no significant differences in other variables between those who experienced maternal and infant adverse events and those who did not (Table 8.1).

**TABLE 8.1:** Comparison between characteristics of pregnancies with and without maternal adverse events.

Parameters	Presence of maternal adverse event		*p*
	No	Yes	
Maternal age (years)	31.0 ± 6.7 31 (16–44)	31.6 ± 5.9 32 (19–43)	0.457^a^
Gestational age at hospital admission (days)	232.3 ± 24.5 246 (177–282)	231.2 ± 22.5 233.5 (180–277)	**0.022^a^**
Gestational age at delivery (days)	244.4 ± 21.7 251.5 (177–282)	235.8 ± 20.9 237.5 (184–277)	**0.002^a^**
Duration from admission to delivery (days)	6.2 ± 10.9 2 (0–67)	4.6 ± 8.2 1.5 (0–42)	0.603^a^
Maternal highest systolic BP (mmHg)	156.1 ± 12.1 153 (140–196)	162.3 ± 14.4 160 (138–201)	**0.003^a^**
Maternal highest diastolic BP (mmHg)	90.8 ± 11.2 90 (57–116)	94.0 ± 10.0 96 (72–120)	0.116^a^
Urine protein/creatinine ratio (mg/kg creatinine)	2314.0 ± 4622.3 499 (52–30372)	2971.5 ± 4211.4 984 (114–18843)	**0.030^a^**
24-h urinary protein (mg/day)	1945.1 ± 3632.7 517 (60–23,421)	2645.5 ± 3206.8 1,128 (142–16,587)	**0.006^a^**
Birth weight (gr)	2339.5 ± 864.5 2492.5 (695–5,670)	2036.6 ± 820.8 1,990 (480–4,175)	**0.028^a^**
1 min Apgar score	7.1 ± 1.7 8 (0–9)	7.2 ± 1.0 7 (2–8)	0.101^a^
5 min Apgar score	8.3 ± 1.8 9 (0–10)	8.5 ± 1.0 9 (3–10)	0.481^a^
Gravida (n)	1 (1–6)	2 (1–6)	0.263^a^
Parity (n)	0 (0–5)	1 (0–5)	0.265^a^
Platelet count (10^3^/ μL)	247.1 ± 71.9 239 (125–449)	223.2 ± 79.1 216.5 (77–428)	**0.039^a^**
Creatinine (mg/dL)	0.54 ± 0.13 0.53 (0.30–0.91)	0.61 ± 0.13 0.61 (0.30–1.00)	**< 0.001^b^**
AST (U/L)	25.0 ± 23.0 19.5 (9–177)	32.0 ± 52.8 23 (12–464)	**0.009^a^**
O_2_ saturation (%)	97.0 ± 0.2 97 (96–98)	96.4 ± 2.1 97 (85–99)	**0.016^a^**
fullPIERS score	0.77 ± 0.78 0.50 (0–4.3)	5.68 ± 15.89 1 (0.1–89.5)	**< 0.001^a^**

^a^Mann Whitney U test;

^b^Independent samples *t*-test. BP, blood pressure; min, minute; AST, aspartate aminotransferase; O_2_, oxygen. Bold values indicate statistical significance.

There were statistically significant differences between those who experienced maternal adverse events and those who did not in terms of preeclampsia type, abdominal pain, maternal complications, cardiovascular events, hematological events, hepatic events, and the presence of other events (*p* < 0.05). The rate of maternal adverse events was significantly higher in those with severe preeclampsia. The rate of maternal adverse events was significantly higher in those with abdominal pain. The rate of maternal adverse events was also significantly higher in the group with maternal complications. The rate of maternal adverse events was statistically significantly higher in those experiencing cardiovascular events, hematological events, hepatic events, and other events. There were no significant differences in other variables between those who experienced maternal adverse events and those who did not (Table 8.2).

**TABLE 8.2:** Comparison between characteristics of pregnancies with and without maternal adverse events (continued).

Parameters		Presence of maternal adverse event		*P*
		No	Yes	
Severe preeclampsia	Yes	79 (53.0%)	70 (47.0%)	**0.041^a^**
	No	16 (80.0%)	4 (20.0%)	
Magnesium prophylaxis	No	21 (70.0%)	9 (30.0%)	0.149^a^
	Yes	75 (53.6%)	65 (46.4%)	
Antenatal steroid prophylaxis	No	58 (61.7%)	36 (38.3%)	0.126^b^
	Yes	38 (50.0%)	38 (50.0%)	
Antihypertensive use	No	59 (62.8%)	35 (37.2%)	0.066^b^
	Yes	37 (48.7%)	39 (51.3%)	
Vaginal bleeding	No	94 (57.7%)	69 (42.3%)	0.242^c^
	Yes	2 (28.6%)	5 (71.4%)	
Abdominal pain	No	91 (59.9%)	61 (40.1%)	**0.019^a^**
	Yes	5 (27.8%)	13 (72.2%)	
Headache/visual disturbance	No	47 (61.8%)	29 (38.2%)	0.204^b^
	Yes	49 (52.1%)	45 (47.9%)	
Proteinuria	No	22 (62.9%)	13 (37.1%)	0.507^a^
	Yes	74 (54.8%)	61 (45.2%)	
Dipstick proteinuria	No	20 (60.6%)	13 (39.4%)	0.735^a^
	Yes	76 (%)	61 (%)	
Maternal complication	No	95 (62.9%)	56 (37.1%)	**<0.001^a^**
	Yes	1 (5.9%)	16 (94.1%)	
Fetal complication	No	90 (56.6%)	69 (43.4%)	1.000^c^
	Yes	5 (55.6%)	4 (44.4%)	
Maternal CNS involvement	No	96 (57.5%)	71 (42.5%)	0.081^c^
	Yes	0 (0%)	3 (100%)	
Maternal CVS involvement	No	95 (78.5%)	26 (21.5%)	**<0.001^a^**
	Yes	1 (2.0%)	48 (98.0%)	
Maternal hematologic involvement	No	96 (62.3%)	58 (37.7%)	**<0.001^a^**
	Yes	0 (0%)	16 (100%)	
Maternal hepatic involvement	No	96 (60.8%)	62 (39.2%)	**<0.001^a^**
	Yes	0 (0%)	12 (100%)	
Maternal renal involvement	No	96 (57.5%)	71 (42.5%)	0.081^c^
	Yes	0 (0%)	3 (100%)	
Placental abruption	No	89 (54.9%)	73 (45.1%)	0.140^c^
	Yes	7 (87.5%)	1 (12.5%)	
Other involvement	No	96 (60.8%)	62 (39.2%)	**<0.001^a^**
	Yes	0 (0%)	12 (100%)	

^a^Yates chi-square test;

^b^Pearson chi-square test;

^c^Fisher chi-square test. CNS, central nervous system; CVS, cardiovascular system. Bold values indicate statistical significance.

There were statistically significant differences between those who experienced and did not experience maternal adverse events in terms of maternal ICU admission, neonatal ICU admission, and chest pain (*p* = 0.011; *p* = 0.002, and *p* = 0.003). The rate of maternal adverse events was significantly higher in those admitted to maternal and neonatal ICUs. Similarly, the rate of maternal adverse events was significantly higher in those with chest pain. There were no significant differences between those who experienced and did not experience maternal adverse events in terms of other variables (Table 8.3).

**TABLE 8.3:** Comparison between characteristics of pregnancies with and without maternal adverse events (continued).

Parameters		Presence of maternal adverse event		*p*
		No	Yes	
Maternal ICU admission	No	95 (59.0%)	66 (41.0%)	**0.011** ^a^
	Yes	1 (11.1%)	8 (88.9%)	
NICU admission	No	59 (67.8%)	28 (32.2%)	**0.002^b^**
	Yes	37 (44.6%)	46 (55.4%)	
Fetal death	No	96 (57.1%)	72 (42.9%)	0.188^a^
	Yes	0 (0%)	2 (100%)	
Infant death	No	93 (56.4%)	72 (43.6%)	1.000^a^
	Yes	3 (60.0%)	2 (40.0%)	
Delivery in 48 h	No	45 (58.4%)	32 (41.6%)	0.637^b^
	Yes	51 (54.8%)	42 (45.2%)	
Delivery in 7 days	No	22 (66.7%)	11 (33.3%)	0.263^c^
	Yes	74 (54.0%)	63 (46.0%)	
Delivery after 7 days	No	74 (54.0%)	63 (46.0%)	0.263^c^
	Yes	22 (66.7%)	11 (33.3%)	
Chest pain	No	96 (58.9%)	67 (41.1%)	**0.003^a^**
	Yes	0 (0%)	7 (100%)	

^a^Fisher chi-square test;

^b^Pearson chi-square test;

^c^Yates chi-square test. ICU, intensive care unit; NICU, neonatal intensive care unit. Bold values indicate statistical significance.

### Discriminative performance of the fullPIERS score in predicting adverse events within 48 h and 7 days

**FIGURE 2:**
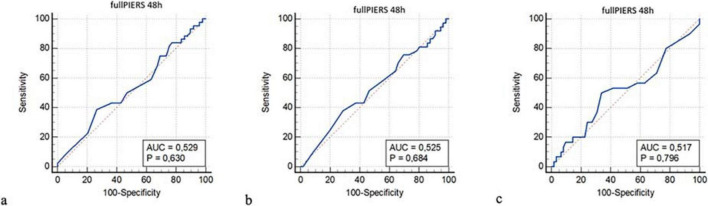
ROC curves for predicting adverse events with in 48 h, **(a)** combined, **(b)** fetal, and **(c)** maternal.

**FIGURE 3:**
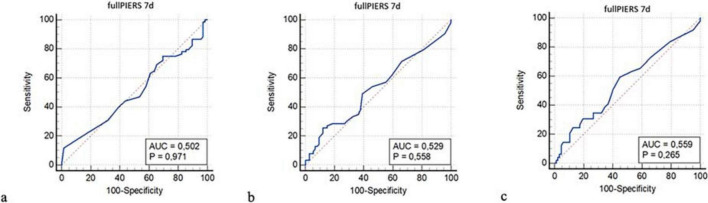
ROC curves for predicting adverse events within 7 days, **(a)** combined, **(b)** fetal, and **(c)** maternal.

Receiver operating characteristic (ROC) curve analysis revealed that the fullPIERS score failed to demonstrate significant discriminative ability for predicting maternal, fetal and maternal-fetal adverse events within 48 h and 7 days (*p* > 0.05) (Figures 2, 3 and Table 9). Consequently, no meaningful optimal cut-off value could be determined for either time point.

**TABLE 9:** ROC Analysis for predicting maternal-fetal, fetal and maternal adverse events within 48 h and 7days.

Adverse event in 48 h	*n*	AUC (95 CI)	*p*	Cut-off	Sensitivity (95% CI)	Specificity (95% CI)
Maternal-fetal	93	0.529 (0.423–0.634)	0.630	≤ 0.3	38.64 (24.4–54.5)	73.47 (58.9–85.1)
Fetal	93	0.525 (0.419–0.630)	0.684	≤ 0.3	37.84 (22.5–55.2)	71.43 (57.8–82.7)
Maternal	93	0.517 (0.411–0.623	0.796	>0.8	50.00 (31.3–68.7)	66.13 (53.0–77.7)
**Adverse event in 7 days**	**n**	**AUC (95 CI)**	***p***	**Cut-off**	**Sensitivity (95% CI)**	**Specificity** (**95% CI)**
Maternal-fetal	137	0.502 (0.415–0.588)	0.971	≤ 0.1	11.76 (5.2–21.9)	98.55 (92.2–100.0)
Fetal	137	0.586 (0.442–0.615)	0.558	> 2	25.40 (15.3–37.9)	87.84 (78.2–94.3)
Maternal	137	0.559 (0.471–0.644)	0.265	> 0.6	59.18 (44.2–73.09	55.17 (44.1–65.9)

The calibration of the model was assessed for maternal, fetal and maternal-fetal adverse events within 48 h and 7 days using the Hosmer-Lemeshow goodness-of-fit test, which indicated an adequate fit (*p* = 0.351, *p* = 0.852 and *p* = 0.500, respectively for 48 h; *p* = 0.266, *p* = 0.134 and *p* = 0.093, respectively for 7 days).

## Discussion

In this retrospective cohort study, we validated the fullPIERS *(full Preeclampsia Integrated Estimate of Risk)* model (4) in a Turkish tertiary-care population and demonstrated that higher fullPIERS scores were significantly associated with adverse maternal, fetal, and combined maternal–neonatal outcomes. The model effectively discriminated high-risk patients using routinely available clinical and laboratory parameters, supporting its applicability in real-world obstetric practice. Our findings are broadly consistent with the original model development study and subsequent external validations, while also highlighting important population-specific considerations relevant to clinical implementation in Türkiye.

The fullPIERS model was originally developed to predict severe maternal complications within 48 h of admission and has since been externally validated in multiple international cohorts (4). Our findings align with prior studies that gestational age at admission, chest pain or dyspnea, oxygen saturation, platelet count, creatinine, and AST levels are strongly associated with adverse maternal outcomes (6, 7). The persistence of these associations in our cohort supports the biological plausibility and robustness of the model across different healthcare systems.

The original fullPIERS model, developed by von Dadelszen et al. (4) and published in The Lancet, demonstrated excellent discriminatory capacity for predicting severe maternal complications within 48 h of admission, with an AUC of 0.88 and sustained performance up to 7 days after assessment. Our findings corroborate the clinical relevance of the same core predictors—gestational age, chest pain or dyspnea, oxygen saturation, platelet count, serum creatinine, and AST levels—which were significantly associated with adverse maternal and neonatal outcomes in our cohort.

Importantly, higher fullPIERS scores in our study were consistently associated with increased rates of maternal, fetal, and combined adverse events, supporting the model’s construct validity in a Turkish population. This aligns with multiple international external validation studies reporting good discrimination across diverse healthcare settings, including Canada, the United Kingdom, the Netherlands, and the United States. Together, these findings reinforce the generalizability of the fullPIERS framework while underscoring the necessity of local validation prior to widespread adoption (8–11).

In our cohort, adverse maternal outcomes were significantly more frequent among women with severe preeclampsia, lower gestational age at admission, elevated systolic blood pressure, renal and hepatic dysfunction, thrombocytopenia, and reduced oxygen saturation. These findings are consistent with the pathophysiological underpinnings of preeclampsia as a multisystem disorder and mirror those reported in the original PIERS cohort and subsequent validation studies (4, 12).

The strong association between chest pain or dyspnea and adverse maternal outcomes in our study is particularly notable. This symptom is a recognized marker of impending cardiopulmonary compromise and has been consistently retained as a key predictor in the fullPIERS model (4, 6, 13). Our findings support its continued inclusion as a high-value clinical variable, especially in resource-constrained or high-volume settings where rapid risk stratification is essential.

Systematic reviews and meta-analyses have shown that among the many predictive tools evaluated for hypertensive disorders of pregnancy, fullPIERS remains one of the few models with sufficient external validation to enable pooled analysis of maternal outcomes (14, 15). Although fullPIERS was designed primarily for maternal risk prediction, higher scores in our study were also associated with adverse neonatal outcomes, including lower birth weight, increased neonatal intensive care unit admission, and infant mortality. These findings likely reflect shared placental and systemic pathophysiology and suggest that maternal risk stratification may have indirect value for anticipating neonatal morbidity (4, 16, 17). The observed association between fullPIERS scores and neonatal outcomes supports the concept that maternal risk stratification may also inform perinatal planning, although neonatal prediction was not the model’s original purpose.

The higher frequency of combined and fetal adverse events observed in preeclamptic pregnancies receiving antenatal corticosteroids appears paradoxical. Closer examination revealed that pregnancies with combined adverse events delivered 18 days earlier, and those with only fetal adverse events delivered 30 days earlier, compared to other pregnancies. As adverse events increase with greater prematurity, the higher rates of combined and fetal adverse events in these patients are likely attributable to earlier gestational age at delivery rather than the administration of antenatal corticosteroids.

The increased frequency of combined and fetal adverse events among preeclamptic pregnancies treated with antihypertensive therapy likely indicates greater disease severity. Antihypertensives are typically used in the expectant management of severe preeclampsia with elevated blood pressures, and their use is therefore associated with higher rates of combined and fetal adverse events. This association is primarily driven by earlier delivery and prematurity, rather than a direct effect on maternal outcomes, as timely maternal management mitigates maternal risk.

The rate of adverse events in mothers and babies was significantly lower among those with placental events. Upon closer examination of this finding, placental abruption occurred in 8 patients, and only one of these experienced intrauterine fetal demise. Among these cases, only one patient had elevated hepatic enzyme levels. As a tertiary referral center, patients with abruption were managed promptly, which may explain the seemingly paradoxical lower rate of adverse events observed in this group.

Unlike the original PIERS study, no significant differences in fullPIERS scores were observed according to delivery within 48 h or 7 days. To assess the potential impact of gestational age, an additional subgroup analysis restricted to pregnancies below 37 weeks of gestation was conducted; nevertheless, fullPIERS scores remained comparable across delivery timing groups. This discrepancy may reflect institution-specific management strategies, earlier intervention thresholds, heterogeneous disease progression or differences in expectant management practices (4). Recent multinational studies evaluating the consecutive or longitudinal use of fullPIERS suggest that, while discrimination may persist, calibration can deteriorate over time, particularly when used repeatedly beyond the initial admission period. This underscores the importance of using fullPIERS as an adjunct to, rather than a replacement for, clinical judgment (8, 13, 16, 18, 19).

Our findings are concordant with prior external validations, which demonstrated good discrimination of severe maternal outcomes using the same core predictors. The consistency of these associations across diverse populations reinforces the generalizability of the fullPIERS model, although variations in absolute risk highlight the importance of local validation. These results emphasize that external validation of the fullPIERS model should be performed in all populations before widespread clinical implementation, ensuring that predictive performance is maintained in different healthcare settings and patient demographics.

Future research should focus on prospective validation, assessment of calibration and decision curve utility, and potential enhancement of the model through integration with angiogenic biomarkers such as placental growth factors *(PIGF)*, which have shown promise in improving risk stratification but require further validation.

This study presented the first external validation of the fullPIERS model in a Turkish population with preeclampsia and demonstrates that higher predicted risk is associated with adverse maternal, fetal, and combined outcomes. The model performed consistently with international validation studies despite differences in population characteristics and clinical practice. Strengths of this study include the first validation of fullPIERS in Türkiye and a comprehensive assessment of maternal and neonatal outcomes. Limitations include its retrospective design, and single center setting. The principal strength of this study is its contribution to geographic validation of fullPIERS in a previously unstudied population. Prospective studies evaluating clinical decision-making, calibration, and integration with angiogenic biomarkers are warranted to optimize risk prediction across diverse settings.

## Conclusion

The fullPIERS model captures key pathophysiological features of severe preeclampsia and demonstrates robust risk discrimination in a Turkish cohort, supporting its broader application in cardiovascular risk-oriented obstetric care. Our findings support the continued use of interpretable, clinically grounded prediction models in hypertensive disorders of pregnancy. While emerging machine-learning approaches may improve discrimination, models such as fullPIERS offer transparency and immediate clinical applicability.

The fullPIERS model performs well in a Turkish tertiary care setting and may support standardized risk assessment for women with preeclampsia, provided local validation and clinical context are taken into account. The fullPIERS model demonstrates good discriminatory performance for adverse outcomes in a Turkish preeclamptic population and represents a valuable adjunct to clinical assessment in tertiary obstetric care.

## Data Availability

The raw data supporting the conclusions of this article will be made available by the authors, without undue reservation.

## Footnotes

1
https://pre-empt.obgyn.ubc.ca/home-page/past-projects/fullPIERS/
